# Time to initiation of antenatal care visit and its predictors among reproductive age women in Ethiopia: Gompertz inverse Gaussian shared frailty model

**DOI:** 10.3389/fgwh.2023.917895

**Published:** 2023-10-03

**Authors:** Daniel Gashaneh Belay, Melaku Birhanu Alemu, Fantu Mamo Aragaw, Melaku Hunie Asratie

**Affiliations:** ^1^ Curtin School of Population Health, Curtin University, Perth, WA, Australia; ^2^ Department of Epidemiology and Biostatistics, Institute of Public Health, College of Medicine and Health Sciences, University of Gondar, Gondar, Ethiopia; ^3^ Department of Human Anatomy, College of Medicine and Health Sciences, University of Gondar, Gondar, Ethiopia; ^4^ Department of Health Systems and Policy, Institute of Public Health, College of Medicine and Health Sciences, University of Gondar, Gondar, Ethiopia; ^5^ Department of Women’s and Family Health, School of Midwifery, College of Medicine and Health Sciences, University of Gondar, Gondar, Ethiopia

**Keywords:** antenatal care visit, maternal health, survival analysis, shared frailty, Ethiopia

## Abstract

**Background:**

Early initiation of antenatal care (ANC) is essential for the early detection of pregnancy-related problems and unfavorable pregnancy outcomes. However, a significant number of mothers do not initiate ANC at the recommended time. Therefore, this study aimed to determine the median time of ANC initiation and its predictors among reproductive-age women in Ethiopia.

**Methods:**

We used the Ethiopian Demographic and Health Survey (EDHS) 2016 data set. The proportional hazard assumption was assessed using Schoenfeld residual test and log–log plot. A life table was used to determine the median survival time (time of ANC initiation). The Gompertz inverse Gaussian shared frailty model was the best-fitting model for identifying the predictors for the early initiation of ANC booking. Finally, the adjusted hazard ratio (AHR) with a 95% confidence interval (CI) was used to determine the significance of predictors.

**Results:**

A total of 7,501 reproductive-aged women gave recent birth in the last 5 years preceding the survey. Nearly three in five women [61.95% (95% CI: 60.85–63.04%)] booked their first ANC visit with a median time of 4.4 months. Women who attended primary education (AHR = 1.10, 95% CI: 1.01–1.20), secondary and above (AHR = 1.26, 95% CI: 1.11–1.44), media exposure (AHR = 1.07, 95% CI: 1.00–1.16), rich wealthy (AHR = 1.17, 95% CI: 1.06–1.30), grand multiparous (AHR = 0.82, 95% CI: 0.72–0.93), unwanted pregnancy (AHR = 0.88, 95% CI: 0.81–0.96), small periphery region (AHR = 0.58, 95% CI: 0.51–0.67), and rural residence (AHR = 0.86, 95% CI: 0.75–0.99) were significantly associated with first ANC visit.

**Conclusion:**

According to this study, a significant number of women missed their first ANC visit. The education status of women, place of residence, region, wealth index, media exposure, unintended pregnancy, and multi-parity were significantly associated with the time of initiation of the first ANC visit. Therefore, policymakers should focus on improving the socioeconomic status (education, media coverage, and wealth) of reproductive-aged women by prioritizing women who live in small periphery regions and rural residences to improve the early initiation of ANC.

## Background

Maternal and child health issues are major public health concerns globally. Maternal and neonatal mortality is unacceptably high with more than one woman dying every 2 min in 2017. Disproportionately, more than 95% of maternal and neonatal deaths occur in low and lower-middle-income countries (1–3). Sub-Saharan Africa takes the lion's share of mortality accounting for more than half of the global burdens, where the maternity continuum of care was scarcely used (1, 4). Ethiopian women have a 21 per 1,000 women lifetime risk for death related to pregnancy with a maternal mortality ratio of 412 per 100,000 live births (5).

The United Nations (UN) Sustainable Development Goal (SDG) sets an objective to reduce maternal mortality to 70 per 100,000 by the year, 2030 with no country falling short more than double this target (1). Providing sustainable and quality maternal care services during pregnancy, childbirth, and the postnatal period can reduce more than two-thirds of maternal and newborn deaths (6, 7). Women who received professional care had a 16% and 24% lower likelihood of losing their baby and experiencing preterm birth, respectively (8). Globally, providing maternity and neonatal continuum of care could prevent approximately half a million neonatal and 3–4 million maternal mortalities (2, 9). The Ministry of Health-Ethiopia (MoH-E) is developing a strategy envisioned to end preventable maternal deaths by 2035 (10) although it looks impossible as evidence points out that maternal mortality is high in the 2016 Ethiopian Demographic and Health Survey (EDHS) (5, 11).

Antenatal care (ANC) services were started across the globe to reduce maternal and neonatal mortality by increasing skilled birth attendance and institutional delivery rate (12–15). Early ANC is defined as the booking of all WHO-recommended services before 16 weeks of gestation, which is vital for the health of both the mother and the neonate (16). The timing of the first ANC visit is very important for subsequent maternal and neonatal care service utilization, which reduces maternal and neonatal mortalities significantly (17–20).

Sociodemographic factors such as parity, education, and wealth status are significantly associated with the time of ANC booking in Pakistan (16). Being a rural residence, married, employed occupation, unplanned pregnancy, and first pregnancy all had a significant impact on the late first ANC initiation (18). However, there is no evidence of the median time of ANC booking among pregnant women in Ethiopia. Therefore, this study aimed to assess the survival time to book the first ANC visit and to identify its possible predictors among pregnant women in Ethiopia. Based on the findings reported from the study, policymakers and stakeholders may be able to develop policies and strategies and design intervention programs to improve maternal care.

## Methodology

### Study design and data source and populations

The study used population-based cross-sectional survey data from EDHS 2016. Ethiopia is an East African country with the second largest population in Africa. Administratively, Ethiopia is federally decentralized into nine regions [Afar, Amhara, Benishangul-Gumuz, Gambela, Harari, Oromia, Somali, Southern Nations, Nationalities, and People's Region (SNNPR), and Tigray] and two administrative cities (Addis Ababa and Dire-Dawa). The EDHS employed a stratified two-stage cluster sampling technique selected in two stages using the 2007 Population and Housing Census (PHC) as a sampling frame. Stratification was achieved by separating each region into urban and rural areas. In the first stage, enumeration areas (EAs) were selected with probability selection proportional to the EA size, and in the second stage, households were systematically selected. The study design and setting are described in detail elsewhere (21).

The study population consisted of women who gave recent birth in the last 5 years preceding the survey. A total of 46 women who responded that they did not know the timing and number of their first ANC visit were excluded from the analysis. Finally, a total weighted sample of 7,501 reproductive-age women was included in the analysis.

### Study variables

The outcome variable of the study is the time between the date of pregnancy of the women and their first ANC visit, which is measured in months. A woman is considered as an event (had her first ANC visit) if she booked WHO-recommended services during her gestational time; otherwise, she is censored. The WHO-recommended services during pregnancies are (1) blood pressure measurements for detecting pre-eclampsia, (2) blood tests for infection and anemia, (3) urine tests for detecting bacteriuria and proteinuria, (4) counseling about the danger signs of pregnancy, (5) provision of iron supplements, and (6) provision of nutritional counseling (22, 23).

Time is defined as the time in months from conception of pregnancy up to the first ANC visit.

Survival time is defined as the time duration of the mother surpassing without the first ANC contact in months.

Failure time is defined as the time in months when the mother gets her first ANC care.

The independent variables considered for this study were categorized as sociodemographic variables such as the age of the mother, marital status, maternal education, education status of the husband, place of residence, household head wealth index, media exposure, pregnancy-related factors such as parity, pregnancy desire, terminated pregnancy, and health facility–related factors such as distance from the health facility, and health insurance coverage.

### Data processing and analysis

The data were accessed in Stata format after registering as an authorized user. We weighed the data as per the recommendation of the major Demographic and Health Survey (DHS). Stata 14 was used for data clearance and analysis. The data were weighted using sampling weight before any statistical analysis to restore the representativeness of the survey. The data clearance and descriptive and summary statistics were conducted using Stata version 14 software. Since the EDHS data have a hierarchical structure where pregnant women are nested within a cluster/EA, the assumption of independent observations and equal variance across the clusters is violated. The random effect of the survival model was checked to assess the clustering effect, and the theta parameter (variance) was used to assess whether there was any significant clustering (24). It showed whether or not there was unobserved heterogeneity or shared frailty that needed to be considered to get a reliable estimate.

Schoenfeld residual test, log–log plot, and Kaplan–Meier and predicted survival plots were applied to check the proportional hazard (PH) assumptions. The log-likelihood ratio test, deviance (−2LL), and Akaike information and criteria (AIC) were applied for model selection. A model with the highest values of log-likelihood and the lowest value of AIC was the best-fitting model. Deviance, AIC, and Cox–Snell residual graph showed that the Gompertz inverse Gaussian shared frailty model had the lowest value and the closest graph to the bisector, which was the best-fitting model for the data (25).

A variable with a *p*-value less than 0.20 in the univariable Gompertz inverse Gaussian shared frailty analysis was included in the multivariable analysis. In the multivariable analysis, the adjusted hazard ratio (AHR) with 95% confidence interval (CI) was used to declare significant predictors for time to first ANC booking. The AHR is the simultaneous inclusion of multiple variables while adjusting for their potential confounding effects. It represents the hazard ratio for the exposure of interest, adjusted for the effects of other variables in the model.

## Result

### Characteristics of the study population

A total of 7,501 reproductive-age women were included in this study, of whom more than half of the mothers were in the age group 25–34 years (55.70%). Most of the study participants [6,934 (92.45%)] were married, and nearly two-third [4,721 (62.94%)] had no formal education (Table 1).

**Table 1:** Characteristics of the study population in Ethiopia, 2016 EDHS.

Variables	Categories	Weighted frequency	Percentage (%)
Maternal age (years)	15–24	1,780	23.73
	25–34	4,178	55.70
	35–49	1,544	20.57
Maternal education	No education	4,721	62.94
	Primary education	2,136	28.00
	Secondary and above	645	8.59
Husband education	No education	3,321	47.29
	Primary education	2,719	39.00
	Secondary and above	983	14.00
Head of household	Male	6,405	85.39
	Female	1,096	14.61
Media exposure	No	4,914	65.52
	Yes	2,586	34.48
Marital status	Not married	566	7.55
	Married	6,934	92.45
Wealth index	Poor	3,271	43.61
	Middle	1,563	20.84
	Rich	2,666	35.55
Insurance covered	No	7,189	95.85
	Yes	312	4.15
Parity	Primiparous	1,408	18.77
	Multiparous	3,161	42.14
	Grand multiparous	2,932	39.09
Terminated pregnancy	No	6,834	91.11
	Yes	667	8.89
Child wantedness	Wanted	6,639	93.57
	Unwanted	456	93.57
Residence	Urban	1,779	23.73
	Rural	4,178	55.70
Distance from HF	Big problem	1,543	20.57
	Not a big problem	3,135	41.79
Region	Metropolis	245	3.27
	Large central	6,821	90.94
	Small periphery	434.7	5.80

HF; Health facility.

### The median time for initiation of the first ANC visit

**Figure 1:**
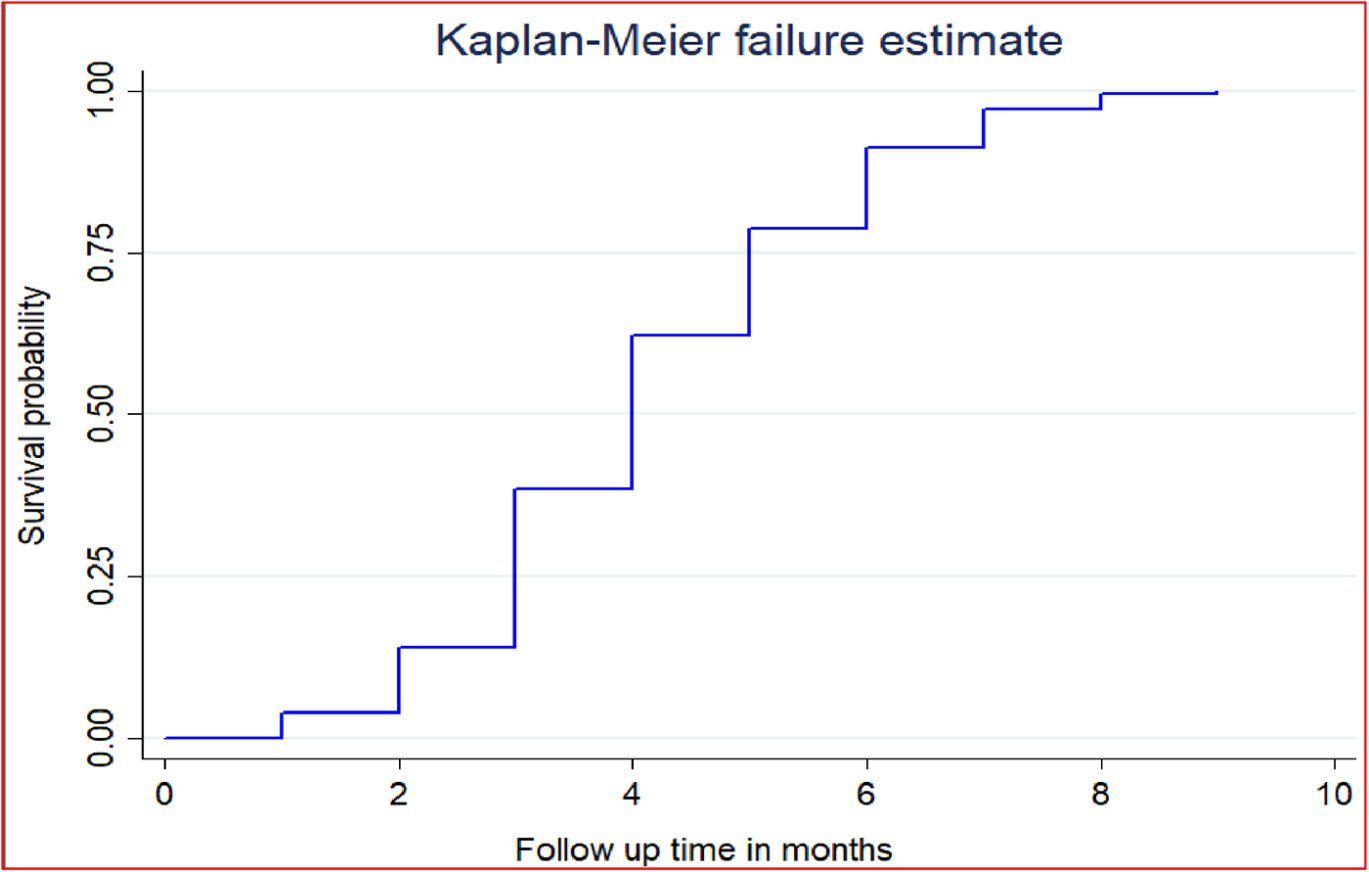
The overall Kaplan–Meier failure curve of initiation of first antenatal care visits in Ethiopia in 2016.

Of the total studied women, 4,701 (61.95%) initiated ANC visits from skilled health personnel, whereas the remaining 2,800 (38.05%) had no ANC visits (they were censored) during the follow-up time. Of those who had ANC, only 62.67% (95% CI: 60.95%–64.35%) of the pregnant women initiated their first ANC visits timely (within 16 weeks of gestational age). Of the total pregnant women, only 35.12% (95% CI: 34.06%–36.20%) initiated their first ANC visits timely. The total follow-up time contributed by all study participants was 19,189 person-years. The overall median survival time (the time when half of the pregnant women were found without booking their first ANC) was 4.4 months. The median survival time varies according to the characteristics of the respondents. The median survival time, for example, in urban areas was 4.0 months, whereas in rural areas (Figure 1).

### Predictors of first ANC visit among women in Ethiopia

#### Comparisons of the survival functions of the first ANC visit for different categorical variables

**Figure 2:**
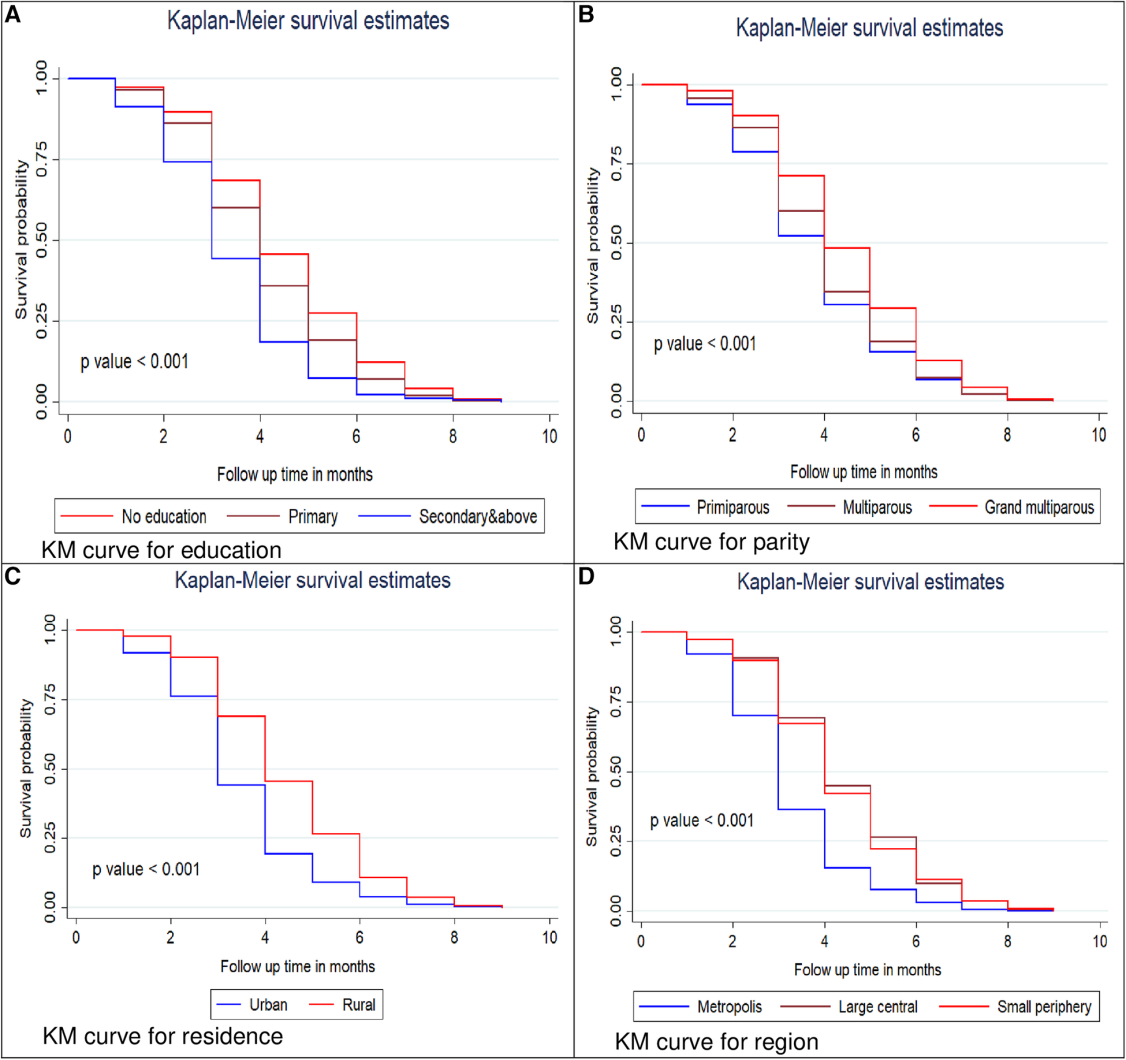
Kaplan–Meier survival curves and log rank tests of initiation of first ANC visits by women education status (**A**), parity (**B**), residence (**C**) and region (**D**) in Ethiopia, 2016.

The log-rank test and the Kaplan–Meier survival function were used to determine the differences in key variables at the baseline among different categories. The Kaplan–Meier survival function was constructed for different categorical variables. In general, the pattern of the survivorship function lying above another indicated that the group defined by the upper curve (red color) had a longer survival (short time failure) than that of the group defined by the lower curve (blue color). Based on this, in our study, rural residents have longer survival than urban residents at a log-rank *p*-value of <0.001. The significance of the graphically observed difference was assessed by log-rank test, and it is indicated in the *p-*value of the respective figures (Figure 2).

#### Model diagnostics and comparison

The Schoenfeld residuals test was used to assess the PH assumption, with results showing that a *p*-value of <0.001 with a chi-square value of 76.06 is significant. This smallest *p*-value is evidence to contradict the PH assumption**.** Therefore, a parametric type of model should be fitted. Based on deviance, AIC, and Cox–Snell residual test, the shared frailty model with Gompertz distribution and inverse Gaussian frailty was most efficient, because it had the lowest deviance and AIC value (Table 2).

**Table 2:** Model diagnostics and comparison for time to initiation of first antenatal care visit and predictors among reproductive-age women in Ethiopia.

Models	Distribution	Frailty	Theta	AIC	BIC	Deviance (−2LL)	LR test of theta
Shared frailty	Gompertz	Gamma	0.33	9,452	9,608	9,776	120
Shared frailty	Gompertz	Inverse Gaussian	0.37	9,447	9,603	9,772	124
Shared frailty	Exponential	Gamma	0.30	9,997	10,140	9,952	106
Shared frailty	Exponential	Inverse Gaussian	0.34	9,992	10,140	9,948	110
Shared frailty	Weibull	Gamma	0.30	9,999	10,150	9,952	105
Shared frailty	Weibull	Inverse Gaussian	0.34	9,994	10,150	9,948	109
Shared frailty	Log-normal	Gamma	0.28	10,340	10,490	10,296	94
Shared frailty	Log-normal	Inverse Gaussian	0.30	10,330	10,490	10,292	98
Shared frailty	Log–log	Gamma	0.29	10,150	10,310	10,112	99
Shared frailty	Log–log	Inverse Gaussian	0.31	10,150	10,310	10,108	102

LR; Likelihood ratio.

In the Gompertz inverse Gaussian shared frailty model, the variables with a *p*-value of <0.2 in the bi-variable analysis were considered for multivariable analysis. Based on these, the variables such as place of residence, maternal education, partner education, wealth index, parity, wanted last pregnancy, and media exposure and residence were significant predictors of the initiation of the first ANC visit in the multivariable analysis.

Women living in rural residences have a 14% lower hazard of initiating their first ANC visits than those living in urban residences (AHR = 0.86, 95% CI: 0.75–0.99). The hazard of initiating the first ANC visit among women who have primary and secondary and higher education is 1.10 and 1.26 times higher than no formal education (AHR = 1.10, 95% CI: 1.01–1.20) and (AHR = 1.26, 95% CI: 1.11–1.44), respectively. The hazard of initiating the first ANC visit among women whose husbands have primary and secondary and higher education is 1.17 and 1.32 times higher than those who had no education (AHR = 1.17, 95% CI: 1.04–1.22) and (AHR = 1.32, 95% CI: 1.12–1.39), respectively. Women who have media exposure have a 1.07 times higher hazard of having their first ANC visits than that in women who have no media exposure (AHR = 1.07, 95% CI: 1.00–1.16). The hazard of initiating the first ANC visit among women who have a rich wealth index is 1.17 times higher than that in those having a poor wealth index (AHR = 1.17, 95% CI: 1.06–1.30). Women who are grand multiparous have an 18% lower hazard of initiating their first ANC visit than that in those primiparous (AHR = 0.82, 95% CI: 0.72–0.93). The hazard of having the first ANC visit among women who had an unwanted last pregnancy was decreased by 18% as compared to that in those with wanted pregnancy (AHR = 0.88, 95% CI: 0.81–0.96)**.** Women who are living in large central and small periphery regions have a 42% decrease in the hazard of initiating their first ANC visit as compared to that in those living in metropolis cities (AHR = 0.58, 95% CI: 0.51–0.67) (Table 3).

**Table 3:** Shard frailty survival regression analysis of initiation of first antenatal care visit among reproductive-age women in Ethiopia, EDHS 2016 perspective.

Variables	Categories	Event (%) *n* = 4,700 (62%)	Failure (%) *n* = 2,800 (38%)	Crude hazard ratio (95% CI)	Adjusted hazard ratio (95% CI)
Age of women	15–24	1,215 (68.25)	565 (31.75)	1.00	1.00
	25–34	894 (21.38)	1,496 (35.82)	0.97 (0.91–1.05)	1.10 (0.99–1.20)
	35–49	804 (52.14)	738 (47.86)	0.84 (0.76–0.93)*	1.07 (0.93–1.23)
Residence	Urban	859 (90.16)	94 (9.84)	1.00	1.00
	Rural	3,842 (58.67)	2,706 (4,133)	0.48 (0.40–0.59)***	0.86 (0.75–0.99)***
Women education status	No education	2,527 (53.54)	2,193 (46.46)	1.00	1.00
	Primary	1,562 (73.19)	572 (26.81)	1.28 (1.19–1.38) ***	1.10 (1.01–1.20)*
	Secondary and above	610 (94.66)	35 (5.36)	1.88 (1.69–2.06) ***	1.26 (1.11–1.44)**
Partner education status	No education	1,767 (53.2)	1,554 (46.8)	1.00	1.00
	Primary	18,03 (66.34)	915 (33.66)	1.25 (1.16–1.36)***	1.17 (1.04–1.22)*
	Secondary and above	843 (85.76)	140 (14.24)	1.78 (1.62–1.96)***	1.32 (1.12–1.39)**
Marital status	Not married	338 (59.75)	228 (40.25)	1.00	1.00
	Married	4,362 (62.91)	2,572 (37.09)	0.96 (0.86–1.08)	1.19 (0.89–1.59)
Head of household	Male	4,024 (62.84)	2,380 (37.16)	1.00	1.00
	Female	676 (61.68)	420 (38.32)	1.12 (1.04–1.22)*	0.07 (0.98–1.17)
Media exposure	No	2,705 (55.03)	2,210 (44.97)	1.00	1.00
	Yes	1,996 (77.17)	590 (22.83)	1.40 (1.30–1.50)***	1.07 (1.00–1.16)*
Wealth index	Poor	1,706 (52.17)	1,564 (47.83)	1.00	1.00
	Middle	975 (62.41)	588 (37.59)	1.10 (0.99–1.21)	1.06 (0.95–1.17)
	Rich	2,018 (75.70)	648 (24.3)	1.58 (1.46–1.72)***	1.17 (1.06–1.30)***
Insurance covered	No	4,465 (62.11)	2,724 (37.89)	1.001.00	1.00
	Yes	236 (75.59)	76 (24.41)	1.21 (1.03–1.43)*	1.19 (1.01–1.41)*
Parity	Primiparous	10.98 (78.02)	309 (21.98)	1.00	1.00
	Multiparous	2,067 (65.42)	10.93 (34.58)	0.91 (0.84–0.98)*	0.92 (0.85–1.02)
	Grand multiparous	1,535 (52.34)	1,397 (47.66)	0.71 (0.65–0.78)***	0.82 (0.72–0.93)**
Child wantedness	Wanted	3,572 (64.79)	1,941 (35.21)	1.00	1.00
	Unwanted	1,127 (56.77)	859 (43.23)	0.86 (0.79–0.93)**	0.88 (0.81–0.96)**
Distance from HF	Big problem	2,372 (54.34)	1,993 (45.66)	1.00	1.00
	Not a big problem	2,338 (74.27)	806 25.73	1.16 (1.08–1.24)*	1.00 (0.94–1.08)
Region	Metropolis	230 (93.99)	15 (6.01)	1.00	1.00
	Large central	4,248 (62.29)	2,572 (37.71)	0.43 (0.38–0.48)**	0.58 (0.50–0.66)***
	Small periphery	221 (50.91)	213 (49.09)	0.43 (0.38–0.491)**	0.58 (0.51–0.67)***

HF; health facility.

Event = women who booked an ANC; failure = women who did not book an ANC.

* *p*-value < 0.05.

** *p*-value < 0.01.

*** *p*-value < 0.001.

## Discussion

This study was conducted to assess the predictors of initiating the first ANC booking in Ethiopia based on the EDHS 2016 data. According to this study, only 61.95% (95% CI: 60.85%–63.04%) of women had their ANC visits. Of those who had ANC, only 62.67% (95% CI: 60.95%–64.35%) of pregnant women initiated their first ANC visits timely (within 16 weeks of gestational age). Of the total pregnant women, only 35.12% (95% CI: 34.06%–36.20%) of women initiated their first ANC visits timely. Moreover, the overall median survival time (the time when half of the pregnant women were found without booking their first ANC) was 4.4 months. This finding was less than the finding from health centers of Addis Ababa, where 65.6% of women started their ANC visit within 16 weeks of gestation. The discrepancy might be because Addis Ababa is the capital of the country and the community there might have better health awareness than other parts of the country. It could also be due to EDHS covering more remote areas where health institutions could be a major predictor of ANC utilization.

In the Gompertz inverse Gaussian shared frailty model analysis, the education statuses of women and husbands, media exposure, wealth index, wanted child, parity, and place of residence were significantly associated with the time of the first ANC visit.

Women who had formal education had a higher chance of booking their first ANC visit as compared to that in women who had no formal education. This is supported by the findings of the studies conducted in Northern (26) and Northwest Ethiopia (27) and Nigeria (28). Better education status of husbands increases the risk of early ANC visits of women as compared to that of their counterparts. This is supported by evidence from a study conducted in Southern Ethiopia (29), where women with educated husbands had more chance of early ANC visits. This is due to being educated to understand the importance of ANC visits, which encourages them to have early ANC bookings.

Women living in rural residences and small periphery regions had less risk of having initiation of ANC visits compared to that of their counterparts. This finding is supported by findings from Zambia (30) and might be explained by urban women who may have better access to health facilities to have an early booking. A better wealth index increases the chance of first ANC visits as compared to the poor. This is supported by evidence reported from Nigeria (28) and Zambia (30), where better household wealth improves the time for women to have their first ANC visit. This might be explained by women with better wealth may have better transport access and the ability to pay for transport to visit health facilities.

Women with media exposure had an increased risk of initiation of their first ANC visit. This is also in line with other findings from Nigeria (28). This could be justified by those women with better media exposure who had better knowledge about the importance of ANC visits, which encourages them to have early ANC bookings.

Being a grand multipara significantly decreases the risk of initiation of the first ANC visit as compared to primiparous women. This is supported by findings of studies conducted in the United Kingdom (31) where having high parity increases the risk of women having late ANC visits. This might be because those women with primigravida are more sensitive to complications and visit health facilities to have experiences with delivery and other services, whereas the multiparous women adapt the pregnancy and labor so they may not visit the health institution early. Women with unwanted pregnancies had a lower risk of initiation of the first ANC visit as compared to those with unwanted pregnancies in Ethiopia. This is in agreement with the reports of studies conducted in Northwest Ethiopia (32) and Zambia (30), where women with wanted pregnancies had a double risk of early initiation of ANC visits. This might be explained by the women with wanted pregnancies who might have a positive experience and more intention to have a healthy neonate with additional support from husbands or families which will encourage them to have an early ANC visit.

The main strength of this study was the use of weighted nationally representative data with a large sample that makes it representative at national and regional levels. Therefore, it can be generalized to all pregnant women during the study period in Ethiopia. Moreover, this study used a shared frailty model that considered the nested nature of the EDHS data and the variability within the community to get a reliable estimate and standard errors. But it is not free of limitations mainly resulting from the use of secondary data. Since the study includes women who delivered in the last 5 years before the data collection and asked about the essential service she provided, there might be a recall bias for relatively older delivery. Moreover, some important confounders like the health service quality and behavioral factors are missed. In addition, the outcome variable is measured in an integer even though the continuous time survival model is fitted.

## Conclusion

According to this study, only three-fifth of pregnant women booked their first ANC visit. The median survival time for initiation of the first ANC visit is higher than what the WHO recommends. The place of residence, education of women and husbands, wealth index, media exposure, pregnancy wantedness, and multi-parity were significantly associated with the time of the first ANC visit.

Therefore, empowering women through improving education level, access to media, and improvements in wealth status can lead to the early booking of ANC by raising awareness and promoting positive healthcare-seeking behaviors. A priority should be given to women in the periphery regions and rural residences, with targeted interventions designed to overcome barriers and ensure equitable access to ANC services for all women.

## Data Availability

The original contributions presented in the study are included in the article/Supplementary Material, further inquiries can be directed to the corresponding author.
